# Human *Streptococcus suis* Infections in Thailand: Epidemiology, Clinical Features, Genotypes, and Susceptibility

**DOI:** 10.3390/tropicalmed7110359

**Published:** 2022-11-08

**Authors:** Anusak Kerdsin

**Affiliations:** Faculty of Public Health, Kasetsart University, Chalermphrakiat Sakon Nakhon Province Campus, Sakon Nakhon 47000, Thailand; anusak.ke@ku.th

**Keywords:** *Streptococcus suis*, serotype, sequence type, susceptibility, outbreak, Thailand

## Abstract

*Streptococcus suis* is a zoonotic pathogen causing substantial economic losses to the pig industry, as well as being a human health burden due to infections worldwide, especially in Southeast Asia. In Thailand, there was high cumulative incidence in humans during 1987–2021, mostly in males. At least five large outbreaks have been documented after the largest outbreak in China in 2005, which was related to the consumption of raw pork or dishes containing pig’s blood. The major clinical features are sepsis or meningitis, with hearing loss a major complication of *S. suis* disease. Thai human *S. suis* isolates have shown diversity in serotypes and sequence types (STs), with serotype 2 and STs 1 and 104 being major genotypes. *β*-Lactam antibiotics can be used in empirical treatment for human *S. suis* infections; however, intermediate resistance to penicillin has been reported. Reducing *S. suis* incidence in Thailand requires a multidimensional approach, with combined efforts from the government and public health sectors through policy, regulations, education, and active surveillance.

## 1. Introduction

*Streptococcus suis* is a Gram-positive coccus bacterium responsible for major infections in pigs and significant economic losses in the pig industry worldwide. The most important clinical manifestations associated with *S. suis* infection in pigs are meningitis, arthritis, endocarditis, pneumonia, rhinitis, abortion, and vaginitis [1]. It is also an emerging zoonotic pathogen causing serious diseases in humans, including meningitis, sepsis, septic shock, infective endocarditis, and septic arthritis [1,2]. The number of reported human *S. suis* cases has substantially increased, with Southeast Asian countries leading the counts, especially Thailand and Vietnam [1,2,3]. Occupations related to pigs or pork, exposure to pig or pork products, or the consumption of raw pork products are the main risk factors of human infection [1,3].

*S. suis* is an encapsulated pathogen, with the capsular polysaccharide antigens being the basis for classification into serotypes [1]. Among 29 serotypes, serotype 2 is considered the most pathogenic and a frequent cause of human disease worldwide [1]. In Western countries, such as the United Kingdom, Spain, Germany, the Netherlands, Canada, and the United States, as well as in Japan, China, and Hong Kong, most human *S. suis* cases have occurred after occupational exposure involving pig handling among pig farmers, bleeders, abattoir workers, carcass cutting and processing workers, butchers, and cooks [3]. However, in Southeast Asian countries, such as Thailand, Vietnam, and Indonesia, a nontrivial number of human cases has occurred in individuals consuming meals containing raw pork meat, blood, and other related products [3]. Two studies in Thailand showed that *S. suis* human infections were responsible for an estimated loss in productivity-adjusted life years to the gross domestic product of USD11.3 million, which equates to USD36,033 lost per person and out-of-pocket expenses for individuals and their families that averaged USD140 (GBP104 or THB5198) per patient [4,5].

In this review, we focus on *S. suis* infections in Thai humans based on epidemiology, clinical manifestations, genotypes, and susceptibility to antimicrobials. Thailand’s high incidence of human *S. suis* cases can provide information for policy implementation, active surveillance, and prevention of this disease.

## 2. Epidemiology of Human *S. suis* Infections in Thailand

In Thailand, *S. suis* infection was first described in 1987 in Bangkok, with two cases of meningitis [6]. Before the largest outbreak of human *S. suis* infection occurred in Sichuan province, China in 2005 [7], sporadic human cases had been reported in several provinces in Thailand, especially in the north [8,9,10,11,12,13,14]. One outbreak with 10 fatal cases due to septic shock was documented in 2000 in Lamphun province, northern Thailand, well before the largest outbreak occurred in China [14]. That study demonstrated that all cases were healthy men aged 40–49 years who were clustered during the same period and geographic area [14]. All cases had a history of chronic alcohol use and the consumption of raw pork or pig’s blood dishes prior to their illness [14]. Notably, all cases in that study had clinical symptoms similar to the cases in the Sichuan outbreak in China in 2005 [7,14].

After the Sichuan outbreak in China, the importance of this disease has been increasingly recognized in Thailand, as well as in many countries worldwide. Up to the present, five large outbreaks of *S. suis* infections in humans have been documented in Thailand: four of these outbreaks were in the north, whereas a fifth outbreak occurred in the northeast. The first outbreak, comprising 29 laboratory-confirmed cases and 3 deaths, occurred in Phayao province during May 2007 [15]. A second outbreak was recognized in Chiang Mai and Lamphun provinces during June–July 2008, with 44 confirmed cases, 26 suspected cases, and 3 fatal cases with septic shock [16]. The third outbreak was reported in Phetchabun province in April 2010, with 14 confirmed cases, of which 5 were fatal [2]. The fourth outbreak was reported in Uttaradit province in May 2019, with 5 confirmed cases and 18 suspected cases [17]. A fifth outbreak occurred for the first time in northeastern Thailand (Nakhon Ratchasima) in 2021, with 21 confirmed cases, including 2 fatal cases [18]. Microbiological analysis of the outbreak *S. suis* strains showed that the first and second outbreaks were due to serotype 2 with sequence type (ST) 1, while the third outbreak was caused by serotype 2-ST104 and serotype 14-ST105. There was no available information about the serotype and STs in the fourth outbreak. The fifth outbreak was caused by serotype 2 with a new ST (ST1656) belonging to the clonal complex (CC) 233/379 [18].

**Figure 1:**
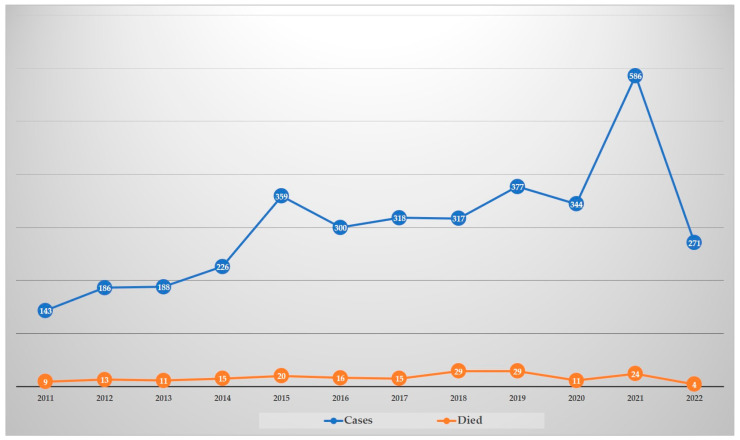
Human *S. suis* cases in Thailand 2011–2022 reported in R506 system by the Bureau of Epidemiology, Department of Disease Control, Ministry of Public Health. For 2022, data are provided only up to September.

National guidelines for human *S. suis* infections are not yet available in Thailand. However, the practice of *S. suis* recruitment in the public health system is conducted using the R506 system (a daily case report of communicable diseases) of the Ministry of Public Health that was initiated after the first large outbreak in 2007. As shown in Figure 1, human cases reported in the system showed an increasing trend during 2011–2021. Although the number of cases dropped in 2022, annual data were only up until September. Notably, these reported cases were submitted by the hospital network where they could identify this bacterium. Thus, misidentification of *S. suis* as other bacteria might have occurred, and this would not have been reported in that system [19,20,21,22]. Therefore, the reported human *S. suis* cases registered in the R506 system may be lower than the real situation.

The national annual crude incidence is 0–0.381 per 100,000 persons [22]. A study in Nakhon Phanom province (northeastern Thailand) documented an annual incidence of 0.1–2.2 cases per 100,000 population for 2006–2012 [22]. This differed from a study in 2010 that estimated human cases to be 730 per year in northern Thailand, with an incidence of 6.2 per 100,000 of the general population [23]. The reasons for these different rates are still unknown, but ethnicity, tribe, cultural behavior, and lifestyle might all influence the *S. suis* infection rate [3].

Three retrospective studies reported that the incidence of *S. suis* disease was high during the rainy season (June–September) [24,25,26]. In contrast, a prospective study in Phayao province, Thailand showed peak incidence in summer (April and May) [23]. In addition, all five outbreaks discussed above occurred in summer. Almost all cases were related to the Songkran Festival (a traditional Thai New Year festival in Thailand) and other harvesting festivals during summer [3].

Several studies revealed that adult age, male sex, alcohol drinking, pig-related occupation or exposure, and raw pork consumption were common risk factors of *S. suis* infections in Thailand [15,27,28]. However, two meningitis cases in children caused by *S. suis* have also been reported in this country [9,29]. The first case was infected with an unknown serotype and ST and the second case was caused by serotype 24 with ST221 [9,29]. Overall, mortality of *S. suis* infections in Thailand is in the range 9.5–19.5% [4,13,24,25,26,30]. Several studies in Thailand have shown that fatal risk factors include septic shock, rapid onset of illness, prolonged bacteremia ≥ 6 days, low serum albumin, high serum total bilirubin, low platelet count, and elevated alanine transaminase [13,26,30]. In particular, septic shock is a strong risk factor, being 22-folds greater than in non-septic shock patients [30].

## 3. Clinical Features of Thai Human *S. suis* Infections

This review collected all reported papers written in either Thai or English that documented *S. suis* infections in Thailand from the available online databases (PubMed, ScienceDirect, Scopus, Google, Thai Index Medicus of Chulalongkorn University, and Siriraj Hospital, Bureau of Epidemiology). Search terms were *S. suis*, human, clinical, Thai, Thailand, outbreak, all years.

In total, 1798 cases from 59 reports were identified [6,8,9,10,11,12,13,14,15,16,17,18,21,22,23,24,25,26,28,29,30,31,32,33,34,35,36,37,38,39,40,41,42,43,44,45,46,47,48,49,50,51,52,53,54,55,56,57,58,59,60,61,62,63,64,65,66,67,68]. Most cases were male (*n* = 1287). Among the 1798 cases, 1052 involved consumption and/or exposure to pig or raw pork products. Septicemia and meningitis were predominant clinical manifestations and hearing loss was a major complication. All this information is summarized in Table 1. According to the retrospective and prospective studies, septicemia/sepsis and meningitis were the most common clinical features found in Thai patients [23,24,25,26].

## 4. Genotypes of Thai Human *S. suis* Strains

As shown in Table 2, for *S. suis* isolated from patients in Thailand, serotype 2 (93.4%) was dominant, followed by serotypes 14 (5.2%), 24 (0.6%), 5 (0.4%), 4 (0.1%), 9 (0.1%), 31 (0.1%), and unencapsulated (0.1%), respectively [21,24,25,32,69]. MLST classified serotype 2 into five CCs: CC1, CC25, CC28, CC104, and CC233/379. Of these, CC1 is a major CC of human *S. suis* infection in this country and ST1 is the main ST in CC1 [25], while serotype 14 was classified to only CC1, with ST105 predominant [25,69]. For serotype 2, ST104, ST25, ST28, and ST233 were the main STs in CC104, CC25, CC28, and CC233/379, respectively. Notably, STs 1 and 104 for serotype 2 are the predominant STs in Thai human infections, and CC104, CC233/379, and CC221/234 are found exclusively in Thailand [24,25,70].

Two retrospective studies revealed that the ST1 strains were more associated with meningitis than the other STs [24,25]. However, the ST104 strains were more associated with non-meningitis, especially sepsis [24]. Differences in clinical manifestations caused by either ST1 or ST104 may have been influenced by genetic backgrounds. Genomic comparison between ST1 and ST104 strains identified that *salK/salR*, the *srtBCD* gene cluster, *revS*, *rgg*, *epf*, and a putative virulence factor SSU0835 (an ABC-type multidrug transport system), with the latter described as being involved in the invasion of porcine brain microvascular endothelial cells, were absent in ST104 [71]. Another study showed ST104 strains failed to develop high levels of meningitis in a mouse model due to low or no production of suilysin by a negligible level of transcription of the *sly* gene and undetectable *sly* promoter activities [72]. That study also illustrated the contribution of suilysin to the development of meningitis by ST1 [72]. These results may explain why ST104 caused less meningitis than sepsis.

Although most cases of Thai human *S. suis* infections had a history of consumption of raw pork dishes, there was no direct evidence or laboratory investigation to confirm or prove the *S. suis* strains in the raw pork dishes that were eaten because none of the raw pork dishes remained after consumption. Indirect investigation was conducted with *S. suis* isolated from slaughterhouse pigs. Three studies showed *S. suis* strains isolated from pigs in Thailand had genotypic profiles of PFGE, RAPD, MLST or combined techniques identical to the *S. suis* strains from humans [73,74,75]. For example, Kerdsin and colleagues (2020) demonstrated that 70.4% of isolates of *S. suis* serotypes 2 and 14 from slaughterhouse pigs revealed STs and PFGE patterns identical to the human isolates [73]. Similarly to Maneerat et al. (2013), the finding showed most of *S. suis* serotype 2 isolates collected from human patients and pigs (diseased and asymptomatic) in different regions of Thailand had the same of ST, RAPD, and virulence-associated gene profile [74], Such indirect evidence suggests the genetic relationships and confirms the possibility of zoonotic transmission of *S. suis* isolates from pigs to humans for certain STs, especially ST1 and ST104, as well as proving that slaughterhouse pigs are a reservoir of pathogenic human *S. suis* strains.

## 5. Antimicrobial Susceptibility

Other studies have revealed that Thai *S. suis* isolates were susceptible to penicillin [13]. This contrasted with Nakaranurack et al. (2017), who reported that 6 out of 11 Thai *S. suis* isolates were intermediately resistance to penicillin, whereas cefotaxime and vancomycin were completely susceptible [35]. However, a study in 2021 demonstrated that 448 *S. suis* isolates recovered from human infections in Thailand had 8.2% intermediate resistance to penicillin, while they were all susceptible to cefepime and ceftriaxone [76]. One study revealed penicillin-resistant *S. suis* with a high minimal inhibitory concentration (MIC) value of >32 μg/mL [77]. Most of the intermediately penicillin-resistant isolates belonged to serotype 2-ST233 [76]. This contrasted with worldwide human *S. suis* isolates showing susceptibility to *β*-lactam antibiotics, including penicillin, ampicillin, amoxicillin, cefotaxime, ceftriaxone, cefepime, meropenem, and imipenem [78,79,80,81,82]. Although there is worldwide usage of *β*-lactams in pigs and humans, almost all human *S. suis* isolates remain susceptible to this antimicrobial class.

Resistance to tetracycline (98.2%), clindamycin (94%), erythromycin (92.4%), and azithromycin (82.6%) with the resistance genes *tet(O)* and *ermB* were the predominant determinant genes of tetracycline and erythromycin (also macrolide-lincosamide–streptogramin B (MLS_B_)) resistance detected in 448 *S. suis* isolates [76]. Resistance to tetracycline appeared common in *S. suis* from human infections worldwide [78,79,80,81,82], whereas the resistance rates to erythromycin were low in Poland, Hong Kong, and Vietnam [78,79,83]. Although *tet(O)* is prevalent in human *S. suis* in Thailand, the *tet(M)* gene is the most widespread in human *S. suis* in China and Vietnam [79,81]. The *ermB* gene was predominant in human isolates in Vietnam and Thailand, while *mefA* was present in Hong Kong [76,79]. This may indicate differences in the local spread of tetracycline and erythromycin-resistance genes among human *S. suis* isolates in different countries or geographical regions.

A recent study investigated an emerging *S. suis* ST1656 belonging to CC233/379 that caused the fifth outbreak in Thailand carried the MLS_B_ resistance gene *ermA* and the oxazolidinone and phenicol resistance gene *optrA* that were found exclusively in the outbreak isolates other than *tet(O)* and *ermB* [18].

## 6. Public Health Control

Kerdsin et al. (2022) mentioned that sociocultural factors, including cultural, religious, and societal behaviors and attitudes associated with the consumption of raw pork or pig’s blood play an important role in human infections [3]. Therefore, effecting a reduction in the human *S. suis* cases in Thailand requires a multidimensional approach involving the government and community sectors. Enforcement is required of meat inspection regulations and hygiene practices at pork processing facilities, as well as conducting food safety campaigns, establishing an educational program on preventing this infection, and reducing behavior regarding the consumption of raw pork or pig’s blood dishes.

Another study in Thailand showed the effectiveness of a food safety campaign [42]. Overall, this campaign led to a marked decrease in the annualized incidence of human *S. suis* disease, from 6.4/100,000 persons in 2010 (before the campaign implementation) to 2.7/100,000 persons in 2011, then to 2.0/100,000 persons in 2012, and finally to 3.5/100,000 persons in 2013 [42]. Finally, early diagnostics in *S. suis* suspected patients using rapid alternative methods rather than traditional culture (a gold standard but slower) could facilitate prompt treatment and reduce the mortality rate, as well as prompt epidemiological investigation [84,85,86,87].

## 7. Conclusions and Perspectives

Thailand has the second-highest number of reported human *S. suis* cases, accounting for 11% of all reported cases worldwide [88]. *S. suis* infections in Thailand had a very high cumulative incidence during 1987–2021, with males being represented in most cases due to them more commonly consuming raw pork or pig’s blood dishes than females. The consumption of raw pork/pig’s blood dishes is common in Thailand, especially in the north. At least five large outbreaks have been documented in Thailand along with the largest reported outbreak in Sichuan, China in 2005, and have been related to the consumption of raw pork/pig’s blood dishes. However, the first large outbreak in Thailand was apparently in 2000. The most common clinical features are sepsis or meningitis, while hearing loss is a major complication of *S. suis* disease.

Thai human *S. suis* isolates have diversified in serotypes and STs, with serotype 2 and STs 1 and 104 being the majority in Thailand. In addition, serotype 14 with ST105 is also prevalent in the country. *β*-Lactam antibiotics can be used in empirical treatment for human *S. suis* infections; however, intermediate resistance to penicillin has been reported. This should be of concern and should be carefully monitored. Thai *S. suis* strains have been reported to be highly resistant to macrolide and tetracycline.

Reducing human *S. suis* disease is Thailand requires a multidimensional approach combining government and public health efforts through policy, regulations, and education, and active community involvement to effect behavioral changes that are evidence-based but culturally sensible and acceptable, along with the implementation in health-care systems of more rapid diagnostics and more relevant screening tools. Most hospital laboratories in Thailand are not able to confirm *S. suis*, and consequently the infection might be misdiagnosed. Improving the capacity of hospital laboratories to identify *S. suis* will aid clinical management, facilitate outbreak detection and response, and facilitate the swift initiation of control measures.

## Figures and Tables

**Table 1:** Characteristics of human *Streptococcus suis* cases in Thailand 1987–2021.

Characteristic	Number of Cases
Total cases	1798
Sex (*n* = 1679)	
Male	1287 (76.6%)
Female	392 (23.4%)
Ratio of male-to-female	3.3:1
Age range (years)	0.15–92
Occupation (*n* = 288)	
Farmer/gardener	153 (53.1%)
Worker/laborer	71 (24.6%)
Employee/officer (government or private)	45 (15.6%)
Butcher	12 (4.2%)
Cook	4 (1.4%)
Housewife	3 (1.1%)
Risk behavior (*n* = 1430)	
Consumption of raw pork/pig’s blood dishes	878 (61.4%)
Exposure to pig/raw pork products	139 (9.7%)
Raw pork product consumption and pig/raw pork exposure	35 (2.5%)
Alcohol drinking/alcoholism	362 (25.3%)
No history of pig/raw pork contact or consumption	16 (1.1%)
Outcome (*n* = 1798)	
Alive	1438 (80%)
Dead	218 (12.1%)
No information	142 (7.9%)
Fatality rate	12.1%
Clinical presentation/type of infection (*n* = 1736)	
Meningitis	709 (40.8%)
Meningitis and septic arthritis	12 (0.7%)
Meningitis and spondylodiscitis	1 (0.06%)
Spondylodiscitis	19 (1.1%)
Septicemia/sepsis	748 (43.1%)
Septic shock	67 (3.8%)
Infective endocarditis	111 (6.4%)
Septic arthritis	51 (2.9%)
Peritonitis	6 (0.4%)
Pneumonia/pulmonary edema	5 (0.3%)
Endophthalmitis	4 (0.2%)
Cellulitis	1 (0.06%)
Acute suppurative thyroiditis	1 (0.06%)
Cholecystitis	1 (0.06%)
Complication (*n* = 432)	
Hearing loss or deafness	324 (75%)
Acute respiratory distress syndrome (ARDS)	23 (5.3%)
Acute renal failure	23 (5.3%)
Disseminated intravascular coagulation (DIC)	19 (4.4%)
Ataxia	12 (2.7%)
Shock	11 (2.5%)
Cranial nerve palsy	5 (1.2%)
Hemiparesis/paralysis	4 (0.9%)
Congestive heart failure	3 (0.7%)
Papilledema	3 (0.7%)
Intracerebral hemorrhage	2 (0.5%)
Subdural empyema	1 (0.2%)
Intervertebral discitis	1 (0.2%)
Stroke	1 (0.2%)
Predisposition (*n* = 455)	
Diabetes mellitus	53 (11.6%)
Hypertension	53 (11.6%)
Heart disease	46 (10.1%)
Spondylitis	27 (5.9%)
Systemic lupus erythematosus (SLE)	21 (4.6%)
Dyslipidemia	16 (3.5%)
Adrenoleukodystrophy (ALD)	16 (3.5%)
Liver cirrhosis	13 (2.9%)
Gout/rheumatoid	4 (0.9%)
Splenectomy	2 (0.4%)
Cancer	2 (0.4%)
HIV/AIDS	2 (0.4%)
Down syndrome	1 (0.2%)
Thyroid	1 (0.2%)
Anemia	1 (0.2%)
Spinal canal stenosis	1 (0.2%)
Unspecified or other underlying diseases	16 (3.5%)
No predisposing factors/healthy	180 (39.6%)

**Table 2:** Distribution of genotypes of *S. suis* isolates from humans.

Serotype	Clonal Complex	Sequence Type	Reference
2	1	1, 11, 105, 126, 144, 298, 337	[18,21,22,23,24,25,29,32,36,37,38,69] https://pubmlst.org/organisms/streptococcus-suis (accessed on 5 October 2022)
	25	25, 102, 103, 380, 381, 395, 515, 516	
	28	28, 382	
	104	101, 104, 391, 392, 393, 512, 513, 514	
	233/379	233, 379, 1656, 1713	
	1687/1688	1687, 1688	
	Singleton	232, 236	
4	94	94	
5	221/234	221	
	Singleton	181, 235	
9	16	16	
14	1	11, 105, 127	
24	221/234	221, 234	
31 (Unencapsulated)	221/234	221	
Unencapsulated serotype 2 or 1/2	28	28	

## Data Availability

Not applicable.
